# Body Mass Index and Prognosis in Ischemic Stroke Patients With Type 2 Diabetes Mellitus

**DOI:** 10.3389/fneur.2019.00563

**Published:** 2019-06-05

**Authors:** Hyungjong Park, Hyung Woo Lee, Joonsang Yoo, Hye Sun Lee, Hyo Suk Nam, Young Dae Kim, Ji Hoe Heo

**Affiliations:** ^1^Department of Neurology, Yonsei University College of Medicine, Seoul, South Korea; ^2^Department of Neurology, Keimyung University School of Medicine, Daegu, South Korea; ^3^Biostatistics Collaboration Unit, Yonsei University College of Medicine, Seoul, South Korea

**Keywords:** ischemic stroke, diabetes mellitus, obesity, body mass index, major adverse cardiac event, mortality

## Abstract

**Background:** Overweight contributes to type 2 diabetes mellitus (T2DM) development. Although the obesity paradox has been suggested in many vascular diseases, little information is available about stroke patients with T2DM. We investigated whether body mass index (BMI) has a differential impact on the incidence of major adverse cardiovascular events (MACE) in patients with ischemic stroke and T2DM.

**Methods:** This retrospective study used a prospective cohort of patients with acute ischemic stroke and included consecutive patients with T2DM after excluding those with active cancer or who died within 1 month of an index stroke. We investigated the long-term risk of MACE (stroke, myocardial infarction, unstable angina, coronary revascularization procedure, and death) according to BMI.

**Results:** Among the 1,338 patients, MACE occurred in 415 patients (31.1%) during a median follow-up of 3.6 years. Compared to the normal weight group, MACE occurred more frequently in the underweight group [adjusted hazard ratio (HR) 1.55, 95% confidence interval (CI): 1.01–2.38], but less frequently in the overweight group (adjusted HR: 0.87, 95% CI: 0.70–1.08) and obese group (adjusted HR: 0.58, 95% CI: 0.41–0.86) group. In analyses of association between BMI and each component of MACE, stroke and cardiovascular mortality indicated an L- and a U-shaped pattern, respectively. However, fatal or non-fatal stroke showed an inverse pattern, and fatal or non-fatal cardiovascular events showed a reversed J-shaped pattern.

**Discussions:** This study showed the overall presence of the obesity paradox in stroke patients with T2DM. However, obese patients had different risks of cardiovascular events and stroke.

## Introduction

Obesity and diabetes mellitus are established risk factors for vascular disease (1). However, leaner adults have higher mortality rates than obese or overweight adults (2–4). This phenomenon has been called the obesity paradox and has been implicated in vascular diseases, including stroke and cardiovascular disease (CVD), as well as chronic diseases, such as hypertension, end stage renal disease, chronic obstructive pulmonary disease, and peripheral artery disease (5–9).

Many patients with type 2 diabetes mellitus (T2DM) are overweight or obese. Overweight is associated with impaired glucose tolerance and insulin resistance, which contribute to the development of T2DM. Due to the pathophysiological relationship between overweight and T2DM, several studies have investigated the association between body mass index (BMI) and mortality in patients with T2DM. Meta-analyses and large cohort studies in patients with T2DM alone showed a U-shaped or J-shaped relationship between BMI and long-term mortality (10–16). In contrast to these T2DM patients, BMI was inversely correlated with mortality in patients with T2DM and acute heart failure (HF) (17). In a prospective cohort study of T2DM patients without known CVD at baseline, overweight or obese patients had a higher rate of cardiac events, such as acute coronary syndrome and HF than those with normal BMI. In the same population, however, the risk of mortality was lower in overweight patients than those with normal BMI (12). These findings suggest that the relationship of BMI with the adverse effects of T2DM may differ depending on the specific disease group. In this study, we investigated whether the incidence of major adverse cardiovascular events (MACE) differs according to BMI in patients with ischemic stroke and T2DM.

## Materials and Methods

### Study Population

This was a retrospective, observational study of prospectively registered patients with ischemic stroke and T2DM included in the Yonsei Stroke Cohort (18). Within 7 days of symptom onset, the cohort enrolled consecutive patients with acute ischemic stroke that have been admitted to the Severance Stroke Center of Yonsei University in South Korea. All patients underwent brain magnetic resonance imaging and/or computerized tomography and cerebral angiography (magnetic resonance angiography, computerized tomography angiography, or digital subtraction angiography). Routine evaluations of patients included standard blood tests, 12-lead electrocardiography, chest radiography, echocardiography, and continuous electrocardiographic monitoring while in the stroke unit or being Holter monitored. Glycated hemoglobin level was routinely assessed in patients with fasting blood sugar ≥ 5.55 mmol/L. All patients were managed according to a standardized protocol and care pathway, which were based on the guidelines.

T2DM was defined based on the American Diabetes Association criteria (19), which include a history of T2DM, current use of hypoglycemic medications or insulin, glycated hemoglobin ≥6.5%, fasting blood glucose level ≥7 mmol/L, or random blood glucose level ≥11.1 mmol/L with typical diabetic symptoms, and no signs of type 1 diabetes. This study was approved by the Institutional Review Board of Yonsei University Health System with a waiver of patients' informed consent due to the retrospective nature of the study.

### Clinical Variables

We collected data for demographics, vascular risk factors for and previous history of stroke, coronary artery occlusive disease, peripheral artery occlusive disease, and chronic kidney disease. Hypertension was defined as systolic blood pressure ≥140 mmHg, diastolic blood pressure ≥90 mmHg, or any history of anti-hypertensive agents. Hyperlipidemia was defined as serum total cholesterol ≥6.21 mmol/L, low-density lipoprotein cholesterol ≥4.14 mmol/L, or history of using lipid-lowering drugs after the diagnosis of hyperlipidemia. Current smoking was defined as a history of smoking any cigarettes within 1 year before admission. We also obtained a history of previously used medication at admission, which included anticoagulants, anti-platelet agents, anti-hypertensive agents, and statins. Laboratory data were also obtained for complete blood counts, lipid profile, initial blood glucose, glycated hemoglobin, blood urea nitrogen, and creatinine. The severity of stroke was assessed using the National Institutes of Health Stroke Scale (NIHSS) at admission. BMI at admission was calculated as weight in kilograms divided by height in meters squared (kg/m^2^).

### Follow-Up and Outcomes

After discharge, we regularly followed up patients at 3 months, 1 year, and every year thereafter. At each follow-up visit, we collected medical data on any cases of cardiovascular events or mortality *via* face-to-face interviews with neurologists or clinical research associates in an outpatient clinic. When the patients missed the visit, we obtained the information from the patient or their families *via* telephone interviews based on a structured questionnaire (20). We obtained necessary data by reviewing the medical records, if available. In addition, we also obtained mortality data from the Korean National Statistical Office (http://www.kostat.go.kr) using death certificates.

The primary outcome was the composite rate of MACE, which include non-fatal or fatal stroke, non-fatal or fatal myocardial infarction (MI), unstable angina, coronary revascularization procedure, and any death. Cardiovascular mortality was defined as any mortality due to MI, other cardiac diseases, or sudden death. Censoring date was December 31, 2013.

### Statistical Analysis

The patients were categorized into four groups according to BMI for the Asian population (underweight: BMI < 18.5 kg/m^2^; normal weight: 18.5 kg/m^2^ ≤ BMI < 23 kg/m^2^; overweight: 23 kg/m^2^ ≤ BMI < 27.5 kg/m^2^; and obese: BMI ≥ 27.5 kg/m^2^) provided by the World Health Organization Expert Consultation panel for appropriate BMI (21). The data were presented as mean ± standard deviation, median (interquartile range), or as number (%), as appropriate. The Shapiro–Wilk test was done to test normality of the continuous variables. The differences between the groups were compared using Kruskal–Wallis test for the continuous variable and a chi-square test or Fisher's exact test for the categorical variable. *Post-hoc* analyses were used for assessing the magnitude of the differences.

The survival curves were determined and plotted using the Kaplan–Meier method, and the group differences in survival time were analyzed using a log-rank test. To determine the independent predictor for MACE, the Cox proportional hazards regression analysis was used and summarized as hazard ratio (HR) and 95% confidential interval (CI). For multivariate analysis, age, sex, initial NIHSS, and variables with *p* < 0.1 in the univariate analyses were entered as covariates. The continuous measure of BMI was used to fit a smooth spline curve to obtain a representation of log HR for each component of MACE and mortality by adjusting the variables that were entered into the multivariate analysis. All tests were two-sided and *p* < 0.05 was considered as statistically significant. R software, 3.3.2 version (R foundation for Statistical Computing, Vienna, Austria) was used for statistical analyses.

## Results

### Characteristics of Patients

**Figure 1:**
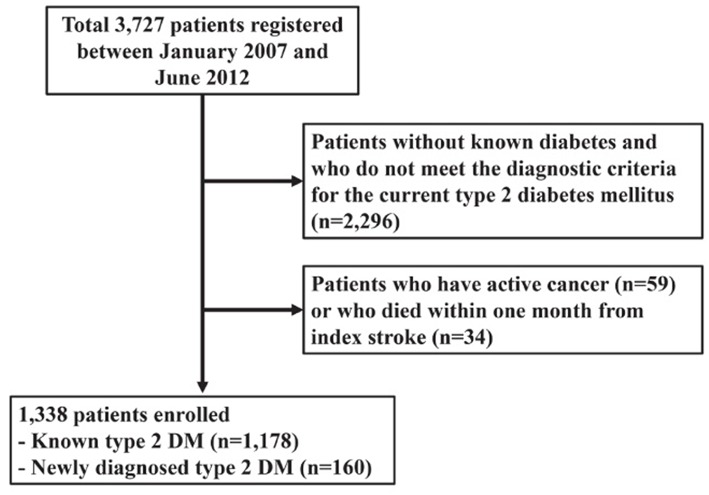
Flow chart of patient selection.

Between January 2007 and July 2013, a total of 3,727 consecutive patients with ischemic stroke were registered. After excluding 2,296 patients without T2DM, we excluded further 59 patients with active cancer (diagnosed or receiving antimitotic treatment within the past 6 months, recurrent, metastatic, or inoperable) (22). Then, we excluded 34 patients who died within 1 month from index stroke because their deaths may have been directly related to the index stroke. Finally, a total of 1,338 patients were included for this study (Figure 1).

The mean age of the patients was 66.6 ± 10.9 years, and 813 were men (60.8%). There were 40 (2.9%) underweight patients, 421 patients (31.5%) with normal weight, 698 overweight patients (52.2%), and 179 obese patients (13.4%). Several variables were different between groups, including age, sex, diastolic blood pressures, initial NIHSS, hemoglobin, blood urea nitrogen, total cholesterol, triglyceride, a history of hypertension, smoking, and chronic kidney disease (Table 1).

**Table 1:** Clinical characteristics of patients according to BMI categories.

	**Underweight (*N* = 40)**	**Normal weight (*N* = 421)**	**Overweight (*N* = 698)**	**Obese (*N* = 179)**	***p***
**DEMOGRAPHICS**					
Age, years	71.0 ± 10.0	68.6 ± 10.9	65.7 ± 10.6	64.6 ± 11.0	0.000^b^, ^c^, ^d^, ^e^
Sex, male	26 (65.0)	244 (58.0)	461 (66.0)	82 (45.8)	0.000^c^, ^d^, ^e^
Systolic blood pressures, mmHg	155.8 ± 31.0	156.4 ± 29.5	156.1 ± 29.4	160.9 ± 31.0	0.261
Diastolic blood pressures, mmHg	78.5 ± 16.6	84.2 ± 17.2	85.2 ± 15.8	87.7 ± 15.0	0.006^a^, ^b^, ^c^, ^e^
Body mass index, kg/m^2^	17.1 ± 1.3	21.3 ± 1.2	24.8 ± 1.2	30.0 ± 3.2	0.000^a^, ^b^, ^c^, ^d^, ^e^, ^f^
Initial NIHSS score	2 [5–10]	2 [4–8]	1 [3–6]	2 [3–6]	0.036^b^, ^d^
**RISK FACTORS**					
Hypertension	29 (72.5)	334 (79.3)	576 (82.5)	160 (89.4)	0.010^c^, ^d^, ^f^
Smoking	6 (15.0)	78 (18.5)	186 (26.6)	27 (15.1)	0.055^d^, ^f^
Hyperlipidemia	7 (17.5)	87 (20.7)	176 (25.2)	51 (28.5)	0.110^e^
PAOD	4 (10.0)	36 (8.6)	41 (5.9)	11 (6.1)	0.296
CAOD	11 (27.5)	120 (28.5)	202 (28.9)	45 (25.1)	0.791
Previous stroke	8 (20.0)	65 (15.4)	108 (15.5)	21 (11.7)	0.485
Atrial fibrillation	10 (25.0)	80 (19.0)	145 (20.8)	29 (16.2)	0.435
Chronic kidney disease	8 (20.0)	74 (17.6)	78 (11.2)	19 (10.6)	0.007^d^, ^e^
**LABORATORY FINDINGS**					
Hemoglobin, g/L	12.8 ± 2.1	13.4 ± 2.0	14.0 ± 2.1	14.0 ± 1.9	0.000^b^, ^c^, ^d^, ^e^
White blood cells, 10^9^/L	8,684.8 ± 2,975.0	8,526.5 ± 3,074.7	8,668.3 ± 3,214.7	8,944.8 ± 3,078.8	0.363
Platelets, 10^9^/L	249.2 ± 79.8	255.7 ± 82.9	249.1 ± 75.2	261.8 ± 81.6	0.126
Blood urea nitrogen, mmol/L	23.4 ± 16.3	19.9 ± 11.9	17.9 ± 9.1	19.4 ± 13.8	0.042^b^, ^c^, ^d^
Creatinine, μmol/L	1.5 ± 1.6	1.3 ± 1.3	1.2 ± 1.2	1.1 ± 1.1	0.326
Total cholesterol, mmol/L	167.6 ± 35.1	177.7 ± 45.7	181.4 ± 46.6	189.1 ± 55.7	0.025^c^, ^e^
Triglyceride, mmol/L	89.0 ± 38.8	128.9 ± 78.6	147.1 ± 100.0	151.1 ± 84.0	0.000^a^, ^b^, ^c^, ^d^, ^e^
HDL-cholesterol, mmol/L	43.1 ± 13.5	40.9 ± 10.6	40.2 ± 10.5	39.6 ± 9.8	0.224
LDL-cholesterol, mmol/L	106.5 ± 32.0	111.1 ± 40.7	111.9 ± 39.8	118.8 ± 43.4	0.155^e^, ^f^
Glycated hemoglobin	7.2 ± 1.4	7.6 ± 1.7	7.6 ± 1.5	7.7 ± 1.5	0.316
Glucose, mmol/L	191.4 ± 80.9	184.3 ± 85.9	189.4 ± 79.7	181.1 ± 74.1	0.356^c^, ^e^
**PREMORBID MEDICATION**					
Antiplatelet agents	17 (42.5)	166 (39.4)	265 (38.0)	66 (36.9)	0.872
Anticoagulants	2 (5.0)	26 (6.2)	37 (5.3)	9 (5.0)	0.916
Statins	9 (22.5)	84 (20.0)	163 (23.4)	41 (22.9)	0.610
Antihypertensive agents	12 (30.0)	142 (33.7)	261 (37.4)	74 (41.3)	0.244

*Data are shown as n (%), mean ± standard deviation, or median [interquartile range]. NIHSS, National Institutes of Health Stroke Scale; PAOD, peripheral artery occlusive disease; CAOD, coronary artery occlusive disease; HDL, high-density lipoprotein; LDL, low-density lipoprotein*.

a *Normal weight vs. Underweight, p < 0.05*.

b *Normal weight vs. Overweight, p < 0.05*.

c *Normal weight vs. Obese, p < 0.05*.

d *Underweight vs. Overweight, p < 0.05*.

e *Underweight vs. Obese, p < 0.05*.

f *Overweight vs. Obese, p < 0.05*.

### Outcomes

During the mean follow-up for 3.6 ± 1.8 years, MACE occurred in 415 patients (31.1%). All-cause death occurred in 351 [26.2%, fatal stroke in 100 (7.5%), fatal MI in 28 (2.1%), and other fatal events in 223 (16.7%)]. Mean annual event rates for MACE were 39.2% in the underweight, 18.0% in the normal weight, 12.0% in the overweight, and 7.5% in the obese group.

**Figure 2:**
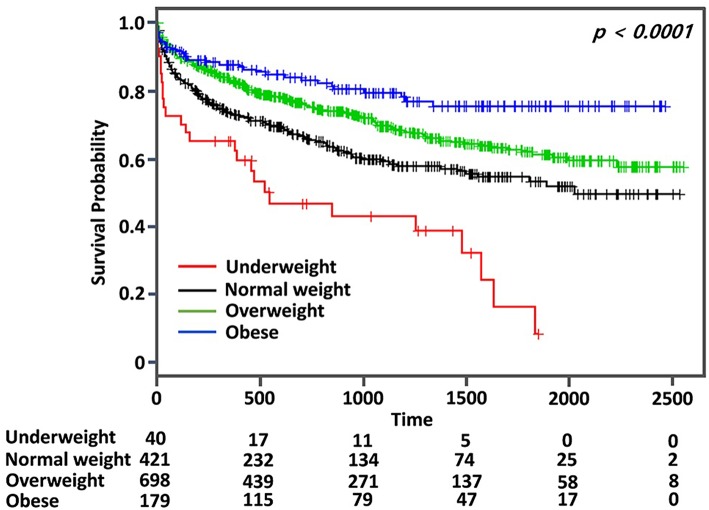
Kaplan–Meier survival analysis for major adverse cardiovascular event (MACE) according to body mass index in patients with ischemic stroke and type 2 diabetes mellitus.

Kaplan–Meier curve analysis showed that MACE occurred more frequently in the normal weight group than the overweight or the obese group, but less frequently than the underweight group (log rank, *p* < 0.001) (Figure 2). After adjustment for age, sex, initial NIHSS, and variables with *p* < 0.10 in the univariate analysis, the obese group showed a significantly lower risk for MACE than the normal weight group (adjusted HR: 0.58, 95% CI: 0.41–0.86, *p* < 0.05) (Table 2). The risk for MACE was significantly higher in the underweight group than the normal weight group (adjusted HR: 1.55, 95% CI: 1.01–2.38, *p* < 0.05) (Table 2).

**Table 2:** Unadjusted and adjusted hazard ratio for MACE of type 2 diabetes patients with acute ischemic stroke according to BMI.

	**Univariate analysis**		**Multivariate analysis**	
	**HR (95% CI)**	***p*-value**	**HR (95% CI)**	***p*-value**
**DEMOGRAPHICS**				
Age, years	1.06 (1.05–1.07)	0.000	1.04 (1.03–1.05)	0.000
Sex, male	0.84 (0.69–0.92)	0.066	1.15 (0.92–1.45)	0.229
Initial NIHSS score	1.10 (1.08–1.11)	0.000	1.1 (1.09–1.11)	0.000
**RISK FACTORS**				
Hypertension	1.31 (0.99–1.72)	0.052		
Smoking	0.73 (0.56–0.93)	0.010	1.22 (0.91–1.63)	0.190
Hyperlipidemia	0.77 (0.60–0.99)	0.032	0.87 (0.65–1.16)	0.333
PAOD	1.64 (1.18–2.27)	0.005	1.15 (0.80–1.63)	0.451
CAOD	1.03 (0.84–1.28)	0.759		
Previous stroke	1.21 (0.95–1.56)	0.138		
Atrial fibrillation	1.87 (1.52–2.32)	0.000	0.97 (0.76–1.23)	0.777
Chronic kidney disease	2.05 (1.62–2.58)	0.000	1.32 (0.96–1.82)	0.088
**LABORATORY FINDINGS**				
Hemoglobin	0.82 (0.78–0.85)	0.000	0.88 (0.84–0.93)	0.000
White blood cells	1.00 (1.00–1.00)	0.007		
Platelets	0.99 (0.99–1.00)	0.052		
Blood urea nitrogen	1.23 (1.02–1.03)	0.000	1.01 (0.99–1.02)	0.146
Creatinine	1.14 (1.08–1.20)	0.000	1.03 (0.94–1.13)	0.273
Total cholesterol	1.00 (0.99–1.00)	0.000	0.98 (0.95–1.02)	0.368
Triglyceride	1.00 (0.99–1.00)	0.000	1.00 (0.99–1.01)	0.515
HDL-cholesterol	0.99 (0.98–1.00)	0.000	1.01 (0.97–1.05)	0.584
LDL-cholesterol	0.99 (0.99–1.00)	0.000	1.02 (0.98–1.06)	0.348
Glycated hemoglobin	0.92 (0.99–0.99)	0.011	1.01 (0.95–1.08)	0.762
**PREMORBID MEDICATION**				
Antiplatelet agents	1.07 (0.88–1.30)	0.503		
Anticoagulants	1.14 (0.77–1.70)	0.517		
Statins	1.10 (0.88–1.38)	0.419		
Antihypertensive agents	1.25 (1.02–1.54)	0.035	1.03 (0.83–1.27)	0.753
**BODY MASS INDEX**		0.000		0.001
Underweight	2.02 (1.34–3.07)	0.001	1.55 (1.01–2.38)	0.046
**Normal weight**	**Reference**		**Reference**	
Overweight	0.70 (0.56–0.86)	0.001	0.87 (0.70–1.08)	0.111
Obese	0.45 (0.31–0.65)	0.000	0.58 (0.41–0.86)	0.001

*HR, hazard ratio; CI, confidence interval; NIHSS, National Institutes of Health Stroke Scale; PAOD, peripheral artery occlusive disease; CAOD, coronary artery occlusive disease; HDL, high-density lipoprotein; LDL, low-density lipoprotein*.

**Figure 3:**
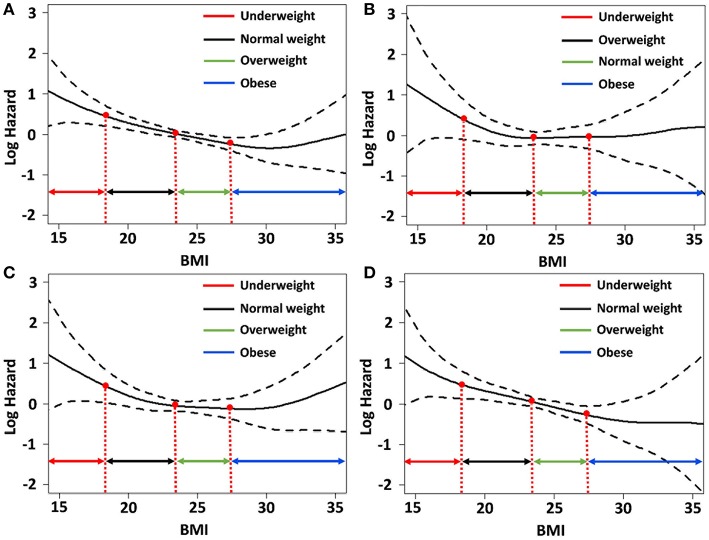
Relative hazards of body mass index (BMI) on **(A)** all-cause mortality; **(B)** fatal stroke; **(C)** fatal cardiovascular events; and **(D)** other-cause mortality after adjusting variables *p* < 0.1 in the univariate Cox regression analysis.

**Figure 4:**
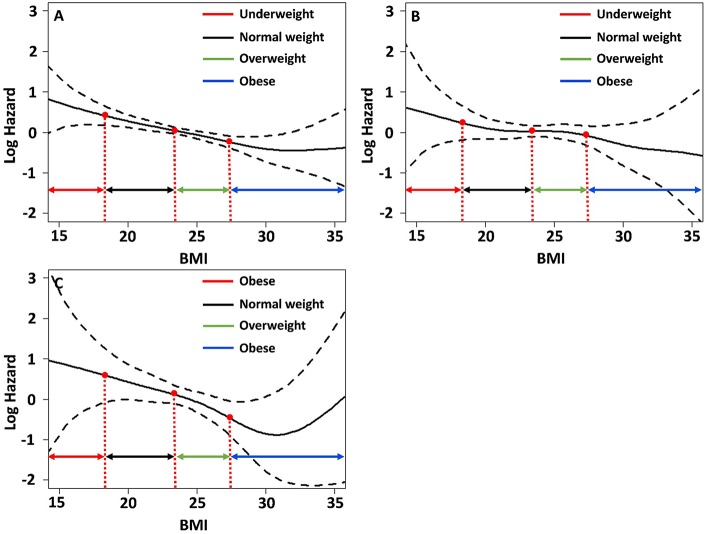
Relative hazards of BMI on **(A)** major adverse cardiovascular events; **(B)** any stroke events; **(C)** any cardiovascular events after adjusting variables *p* < 0.1 in the univariate Cox regression analysis.

We determined the association of BMI with the risk for each component of MACE and mortality using the spline curve of log HR according to BMI as a continuous variable. While the all-cause mortality and cardiovascular mortality showed a U-shaped or a reversed J-shaped pattern, stroke mortality showed an L-shaped pattern. Other-cause mortality (death due to conditions other than CVD or stroke) showed an inverse relationship with BMI (Figure 3). A stroke (fatal or non-fatal stroke) showed a pattern of inverse relationship with BMI. However, any cardiovascular event (fatal or non-fatal event) showed a reversed J-shaped pattern in that the slope of the curve was sharply increased in body weight range belonging to the obese group (Figure 4).

## Discussion

This study showed an inverse relationship between BMI and occurrence of MACE in patients with acute stroke and T2DM. However, the risk for each component of MACE differed according to the BMI. While BMI had an inverse association with other-cause mortality, it had an L-shaped association with stroke mortality, and a U-shaped association with cardiovascular mortality.

Most studies in patients with T2DM alone showed the U-shaped association of BMI with all-cause and cardiovascular mortality (10, 11, 13, 14, 23). However, studies in T2DM patients with comorbidity suggested heterogeneity in mortality according to the specific comorbidity. The J-shaped pattern of mortality was observed in T2DM patients who were free of CVD and cancer (16). In patients with acute MI and T2DM, there were no survival advantages from an elevated BMI (24). While there was U-shaped association in patients with chronic HF and T2DM, there was an inverse association in those with acute HF and T2DM (17). This study also showed that the risk of cardiovascular mortality was higher in obese patients than in overweight patients. However, stroke mortality was not higher in obese patients, which supports the heterogeneity of mortality in T2DM patients according to the comorbidity.

While a majority of previous studies on the obesity paradox have focused on mortality, overweight or obesity may be a risk for vascular events. Therefore, we investigated the occurrence of non-fatal stroke and cardiovascular events as well as fatal events. This analysis showed that the risk for any stroke (fatal or non-fatal) in obese patients decreased and showed an inverse association with BMI. However, the risk of any cardiovascular events (fatal or non-fatal) sharply increased in obese patients and showed a reversed J-shaped pattern. These findings suggest that the risk of stroke and cardiovascular events is different in stroke patients with T2DM and obesity, in that the risk of cardiovascular events may increase and that the risk of stroke may not increase. The reason for the discrepancy in stroke incidences and cardiovascular events in obese patients is uncertain. However, while obesity is a common risk factor for both cardiovascular events and stroke, the attributable risk of obesity is higher for cardiovascular events than stroke (25).

In contrast to obese patients, underweight patients with T2DM and stroke consistently showed poor outcomes in this study. Previous studies also showed poor outcomes in underweight T2DM patients. The patients who have a greater genetic susceptibility to T2DM have a greater chance of developing T2DM at lower BMI, which will consequently lead to a poor prognosis (12). In a study that assessed weight loss after stroke, weight loss > 3 kg was associated with poor outcomes after stroke (26). Muscle power is crucial for mobilization and effective rehabilitation after stroke. Immobilization is associated with various complications and poor outcomes (27, 28). Lean muscle mass in underweight patients might negatively impact their mobility, which could contribute to poor outcome.

This study in stroke patients with T2DM yielded the best outcomes in terms of mortality and MACE in the overweight. However, it remains uncertain whether an intervention for body weight modification should be targeted to remain overweight because our findings and others' are based on observational studies. Current guidelines for the secondary prevention of ischemic stroke recommend weight loss and aerobic exercise (29, 30). Strength training is also beneficial in patients with T2DM as it increases glucose uptake and insulin signaling in skeletal muscles (31). Thus, instead of a uniform weight loss recommendation for ischemic stroke patients with T2DM, an individualized approach depending on weight status may be needed.

There are limitations in our study. First, this study is a retrospective, single-center study in the single ethnic population, which limits the generalization of findings. Second, we simply categorized the patients according to BMI at admission, which does not reflect changes in body weight over time. In addition, this study did not assess the muscle mass, which may be an important factor for prognosis. Third, while it is known that each category of diabetic drug may affect body weight (32), information about diabetic medications before admission was not collected. Lastly, this study may be subjected to the selection bias because a significant portion of screened patients excluded according to the inclusion criteria.

## Conclusions

This study showed the overall presence of the obesity paradox in stroke patients with T2DM. However, obese patients had different risks of cardiovascular events and stroke. Since the risk of cardiovascular events sharply increased in obese patients, we may have to be more attentive to these patients to prevent these events. This could be done by assessing for asymptomatic coronary artery disease, considering that approximately one-third of stroke patients have asymptomatic significant (≥50%) coronary artery disease, and these patients have poor long-term outcomes (20, 33).

## Data Availability

The datasets for this manuscript are not publicly available because This is the registry including patients' information. Although this information was collected after informed consent, this information cannot be publicly available because it contained individuals' information. Requests to access the datasets should be directed to hjpark209042@gmail.com.

## Author Contributions

HP, HWL, JY, HSN, YDK, and JH designed the study, collected data, and drafted the manuscript. HP and HSL performed the statistical analysis. All authors interpreted the data and revised the manuscript.

### Conflict of Interest Statement

The authors declare that the research was conducted in the absence of any commercial or financial relationships that could be construed as a potential conflict of interest.
